# DeWitt County Medical Society

**Published:** 1866-05

**Authors:** C. Goodbrake

**Affiliations:** Sec’y.


### DE WITT COUNTY MEDICAL SOCIETY.

The Society met in annual session, in Clinton, at the office of Dr. Shurtleff, on Monday the 2d day of April, 1866, Dr. Tyler in the Chair.

The minutes of the previous meeting were read and approved.

On motion of Dr. Madden, Dr. Drombolone and Dr. Jno. A. Edmiston were invited to participate in the proceedings of the meeting.

The election of officers being in order, the following gentlemen were elected for the ensuing year: President, Dr. John Wright; Vice-President, Dr. B. K. Shurtleff; Secretary, Dr. C. Goodbrake; Censors, Dr. J. H. Tyler, Dr. R. T. Richards and Dr. T. W. Davis.

Delegates to the Ill. State Medical Society, Dr. R. T. Richards, Dr. B. K. Shurtleff and Dr. T. W. Davis.

Delegates to the American Medical Association, Dr. B. S. Lewis and Dr. T. K. Edmiston.

Dr. Tyler, the retiring President, then delivered an address on the diseases most prevalent in DeWitt county during the past year, for which he received the thanks of the Society.

Dr. John A. Edmiston, of Clinton, and Dr. Charles W. Drombolone, of Marion, having applied for membership, and the Censors having reported favorably, they were unanimously elected members of the Society.

Dr. Z. H. Madden reported a case of intermittent fever, followed by partial paralysis, which called up a discussion, in which most of the members present participated.

The essayists appointed at the last meeting, having failed to fulfill their duty, were continued, and the President also appointed Drs. J. A. Edmiston and C. W. Drombolone to read each an essay at the next meeting.

Cholera was chosen as the subject for discussion at the next meeting, and after the passage of a series of sanitary resolutions, referring to the impending epidemic, the Society adjourned to meet in quarterly session at Clinton, on the 1st Monday of July next, at 10 o'clock A. M., at Dr. Shurtleff's office.

C. GOODBRAKE, *Sec'y.*

